# Analysis of Membrane Behavior of a Normally Closed Microvalve Using a Fluid-Structure Interaction Model

**DOI:** 10.3390/mi8120355

**Published:** 2017-12-06

**Authors:** Guru Prasath Natarajan, Sung-Jin Kim, Chang-Wan Kim

**Affiliations:** Department of Mechanical Engineering, Konkuk University, Seoul 05029, Korea; guruprasatharnav@gmail.com (G.P.N.); yahokim@konkuk.ac.kr (S.-J.K.)

**Keywords:** microvalve, fluid-structure interaction, opening threshold pressure, membrane behavior

## Abstract

In this paper, membrane deflection against fluid flow and opening membrane (threshold) pressure were studied using fluid-structure interaction (FSI) analysis, and compared with experimental data obtained by Jaemin et al. In the current analysis, two different models (I-shaped and V-shaped) were used to perform the FSI simulation. In microvalve modeling, in order to reduce external actuator usage, interconnections are made between two similar microvalves. This typical interconnection creates a pressure distribution in a local environment. Furthermore, to differentiate the volume factor in a microvalve, a length/width (L/W) ratio term was used. Compared with higher- and lower-L/W-ratio models, the higher-L/W model eventually initiates more deflection in a low-pressure regime than the lower-L/W-ratio model. FSI simulations were performed for 4 μL/min, 6 μL/min, 8 μL/min, 10 μL/min, and 12 μL/min flow rates against membrane behavior, and performance evaluations of the microvalves were conducted. It was observed during an FSI simulation that the gate pressure applied to the lower surface deflects the membrane upward, thereby making contact with the wall. Two important parameters (material properties of the structural membrane and the inlet region height) were selected for analysis to evaluate changes in microvalve performance. These results are presented in the current study.

## 1. Introduction

Microfluidics deals with the volumes of fluids in sizes of nanoliters to picoliters [1]. It is a multidisciplinary field that includes engineering [2,3,4,5], chemistry [6,7], biochemistry [8], and biotechnology [1,7]. For various applications, implementing highly integrated microfluidic chips requires consideration of the performance of the chips as well as selecting on-chip components such as pumps, valves, and actuators [9] Among the components, a compact and efficient microvalve is important to control the on-off switching of flows.

Generally, there are two kinds of microvalves: (1) normally open (NO) and (2) normally closed (NC) [10,11]. Many previous works have studied the two types of microvalves in terms of performance and actuation. To study the actuation process of microvalves, some researchers performed experimental analyses or fluid-structure interaction (FSI) simulations to realize the behavior of the membranes of the microvalves. In experimental analyses for large-scale integration and high throughput in fluid manipulation, NC glass microfluidic devices are fabricated using three- and four-layer device topologies, and achieved the parallel activation of the valves and pump [12]. Afterwards, matrix-shaped microfluidic devices were fabricated using parallel channels with the orthogonally crossed elastic material polydimethylsiloxane (PDMS) to produce an automatic deflection of material while applying pressure in the channel. The experimental study was validated by using theoretical modeling [13]. Mohan et al. performed an experimental analysis by fabricating three different shapes of pneumatically actuated microvalves (i.e., valve seats having an I-shape, V-shape, and diagonal shape), and showed that a V-shaped microvalve requires the lowest actuation pressure to open itself [14]. Jaemin et al. fabricated and performed an experimental analysis on I- and V-shaped models (changing the shape using length/width (L/W) ratio terms) in NC microvalve models [11]. This did not require an external actuation system to control the deflection of the membrane, and a low constant opening membrane pressure was achieved.

Several researchers performed FSI analyses on various types of microvalves. Most of them selected a two-way FSI analysis to describe the deformation of the microvalve membrane. Yang and Kao analytically and numerically investigated the deformation of a microelectromechanical system (MEMS) diaphragm valve against the effect of fluid flow [4]. Later, Afrasiab et al. analyzed a valveless microfluidic device actuated by a piezoelectric film using FSI governing equations [5]. Then, finite element method (FEM) and FSI simulations were performed on a pneumatically actuated diaphragm microvalve to study fluid flow and diaphragm deflection [3]. Afterwards, a single-channel microfluidic device was built with a PDMS flexible membrane to examine the membrane deflection and pressure drop in a microfluidic device using both experimental and FSI analyses [2]. In the research of Sun hao et al., a three-dimensional multiphysical simulation on a microfluidic system was performed to analyze the pressure, flow velocity, and temperate distribution [15]. However, there has been little research on the NC microvalve using FSI simulations to study performance and membrane behavior against fluid flow.

In this work, a two-way FSI analysis is performed on an NC microvalve model. This FSI analysis was performed in order to understand the fluid flow inside the microvalve, the membrane deflection against fluid flow, and changes in performance of the microvalve according to changes in parameters. Finally, results from the FSI analysis are compared with experimental data [11]. In particular, various L/W-ratio models of the microvalve are analyzed using the FSI method to evaluate the performance and membrane behavior of the microvalve against fluid flow. Additionally, parameters such as the structural material properties of the microvalve membrane and the inlet region height are selected to check the effects of changes in parameters against performance. The results we obtained in this analysis will be helpful for designing NC microvalves.

## 2. Numerical Modeling of 3D Microvalve

### 2.1. Microvalve Design

**Figure 1:**
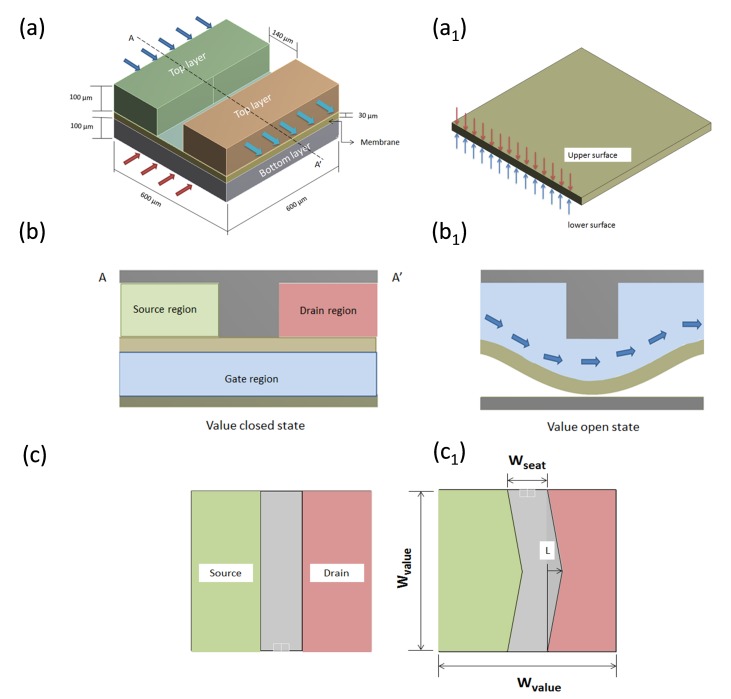
Normally closed (NC) microvalves: (**a**) 3D microvalve, and dimensions of top and bottom actuation chamber; (**a_1_**) Structural membrane; (**b**,**b_1_**) Cross-sectional view of microvalves open and closed structural domain in A-A’ position, and source, drain and gate regions of the valve. Open and closed states of microvalve: (**c**,**c_1_**) top view of microvalve, and measurement of different vertex positions.

Figure 1a illustrates a three-dimensional (3D) microvalve device. This typical microvalve device contains two layers (top and bottom), also called actuation chambers. Jaemin et al. performed a flow analysis inside a microvalve [11]. In that analysis, to avoid external devices (i.e., the pneumatic system, cylinder, and pumps) in controlling membrane fluctuation in the microvalve, two similar NC microvalves were connected in a distinctive manner. This achieved a fluctuating fluid flow and pressure distribution in the interior regions of the microvalve to control the deflection of the structural membrane (shown in Figure 1a_1_). Typically, an NC microvalve design contains source ($P_{S}$), drain ($P_{D}$) and gate ($P_{G}$) regions, as shown in Figure 1b. The source region ($P_{S}$) and drain region ($P_{D}$) are otherwise indicated as the inlet and outlet regions, respectively. The maximum deformation obtained from the microvalve’s membrane is restricted by the height of the drain region.

The microvalve design specification is taken from [11]. These models are used to perform an FSI analysis. The dimensions of the microvalve design and the material properties of the solid and the fluid are listed in Table 1, Table 2 and Table 3, respectively. To achieve a low constant opening membrane pressure, various microvalve model shapes are used, and an FSI analysis is conducted. The generated FSI results are compared with those of an experimental analysis [11], which are shown in the sections below. To change the design and shape of the microvalve, the vertex is varied to several position lengths (L): 0 μm, 50 μm, 100 μm, 150 μm, and 200 μm. This is shown in Figure 1c. Changes in the vertex length of the microvalve models are mentioned using the parameter of the L/W ratio. Further explanation of the L/W-ratio parameter is given below.

### 2.2. L/W Ratio in Microvalve Models

The L/W (length/width) ratio is the change in position of the valve seat and shape in a microvalve design by varying the vertex length (L). In FSI analysis, various L/W-ratio microvalve models are used to understand the behavior of membrane deflection against the fluid flow in the microvalve. The length in the L/W ratio is indicated as the vertex length (L). Width (W) is a constant value, and it is calculated by (($W_{\mathrm{Valve}}-W_{\mathrm{seat}})/2$), where $W_{\mathrm{Valve}}$ and $W_{\mathrm{seat}}$ are the widths of the valve and seat, respectively.

**Figure 2:**
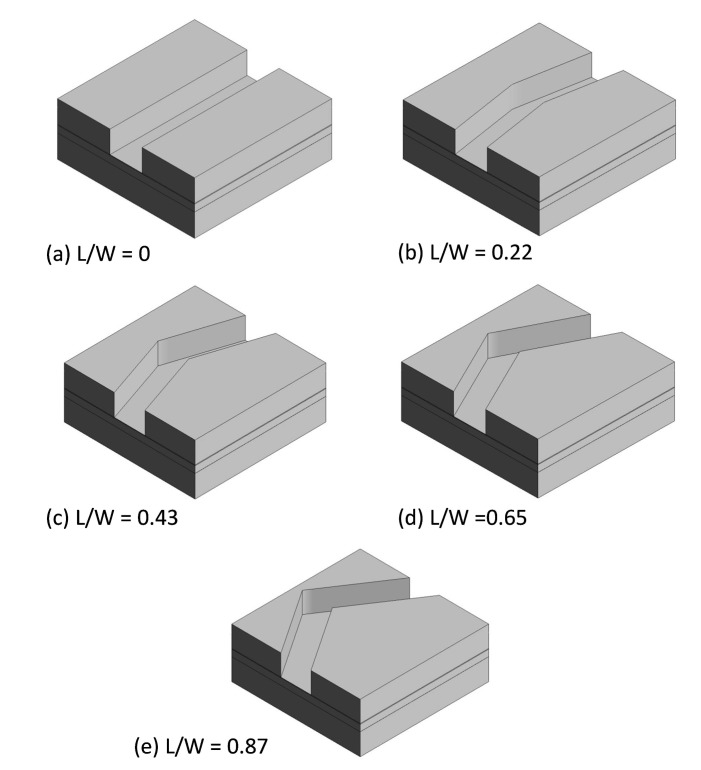
(**a**–**e**) Shows that various shapes of microvalve fluid domain model illustrated with length/width (L/W) ratio.

For example, when calculating the L/W ratio, if the vertex length (L) is 50 μm and width W is 230 μm (constant), the L/W ratio is 0.22. Similarly, in other models, the L/W ratios are calculated according to the above-mentioned method. This calculating procedure for the L/W ratio was inspired by Jaemin et al. [11]. As shown in Figure 2, an increase in the L/W ratio separates the volumes of the source and drain regions. Therefore, in higher-L/W models, the source region has a large volume, and simultaneously the drain region will have a lower volume. The separation in region volumes creates a greater pressure difference in the source region; hence, quick deflection in the structural membrane is achieved.

### 2.3. Operation of Microvalve

**Figure 3:**
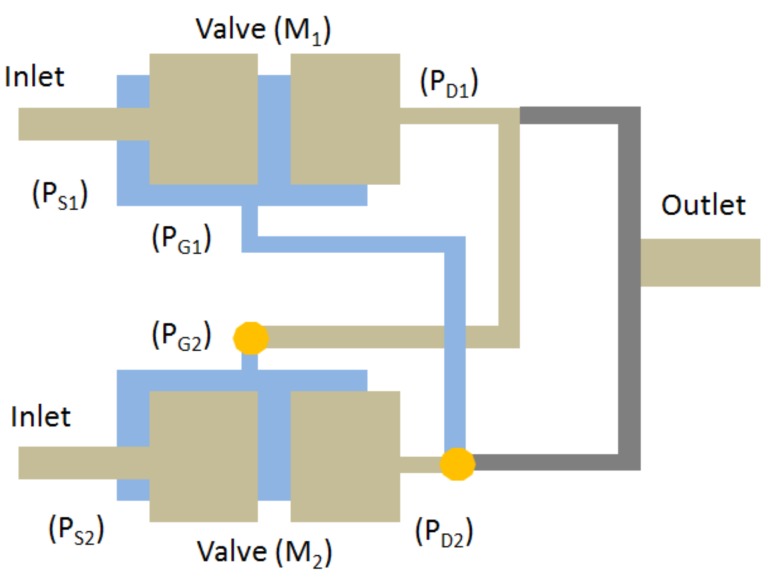
Interconnection of microvalve regions.

In an experimental analysis [11] to achieve a low constant opening threshold pressure, the various regions of two similar microvalves are interconnected. Fluid flow between these different regions creates pressure fluctuations in the microvalve to control the structural membrane deformation. This process is performed with the microvalve in an open and closed state. For example, the source regions (PS) (inlet regions) of two similar microvalves (named $M_{1}$ and $M_{2}$) are connected to the inlet fluid flow. The drain region ($P_{D1}$) outlet of microvalve $M_{1}$ is connected to the gate region ($P_{G2}$) of microvalve $M_{2}$. Similarly, the outlet of microvalve $M_{2}$ is connected to the gate region of microvalve $M_{1}$. This is shown in Figure 3. This way of interconnecting regions creates fluctuations in the fluid flow between the two microvalves.

The inlet is connected to the source regions (PS) of microvalves $M_{1}$ and $M_{2}$. The flow to these microvalves is independent. For example, if the inlet of microvalve $M_{1}$ is open, then the inlet of microvalve $M_{2}$ is closed. If the fluid flow through the input of microvalve $M_{1}$ ($P_{S1}$) creates pressure in the source region, and the developed pressure in that region is applied over the upper surface of the structural membrane. Then, pressures on the surface on the membrane deflect downward and make way for fluid to flow from the inlet ($P_{S1}$) to the outlet ($P_{D1}$). This phenomenon is called the open state of the microvalve. Simultaneously, per the interconnections, the flow from the drain region ($P_{D1}$) outlet is connected to the gate region ($P_{G2}$). This process creates pressure in the gate region ($P_{G2}$) in microvalve $M_{2}$ and forces the membrane to deflect upward. This process is called the closed state of the membrane. This interconnection method operates as a cyclic process. In FSI analysis, a working procedure identical to that above from an experimental analysis is applied. To reduce the computational overhead , just microvalve $M_{1}$ is taken and analysis is performed in complete cycle. The complete cycle takes around 60 s to transform from the open to closed state of a lone microvalve. Therefore, during the analysis, the first 30 s the inlet flow is sent to the top region of the microvalve to perform the open-state process. Similarly, the last 30 s of the flow is sent to the bottom region of the microvalve for the closed-state process. Finally, the performance of microvalve and the behavior of the structural membrane are examined, and the results are given below.

## 3. Fluid-Structure Interaction

In order to study a coupled field analysis of the fluid flow and structural deformation in microfluidic devices, a fluid-structure interaction was performed using commercial ANSYS software (version 16.2, ANSYS, Inc., Canonsburg, PA, USA) [16]. The coupled-field analysis is a multidisciplinary analysis that is associated with two or more fields to solve various engineering problems. This coupling analysis can be conducted using a one-way or two-way method. For the analysis of small structural deformations, the one-way FSI method is more efficient and less time consuming. The two-way FSI coupling method is performed when structural deformations are not in the negligible range. However, if a highly elastic material is used for the structural analysis, then the deformation is large.

In the microvalve FSI analysis, elastic material is used for the structural membrane. This is shown in Table 2. Hence, the elastic material obtains large structural deformations in the membrane. Therefore, the fluid and structure field should couple in the two-way analysis to produce more accurate results for this problem. Thus, a two-way FSI coupling analysis is used to solve microvalve problems in an iterative manner to produce accurate results. In this work, the membrane of the value is modelled as a 3D solid, which facilitates the convergence of the FSI coupling. In order to avoid the excessive element distortion in solid membrane of the value due to large deformation of the FSI interface, the elastic analogy mesh motion method is used [3,17,18].

### 3.1. Governing Equations in FSI

Coupled two-way fluid-structure interaction problems need to consider every field, i.e., the fluid flow, structural deformations, and moving mesh. The governing equation for all these fields is referred to in [3,5,19] and is given below.

#### 3.1.1. Fluid Formulation

The conservation laws of momentum and the continuity equation of a viscous, incompressible flow comprising the Navier-Stokes equation with arbitrary Lagrangian-Eulerian (ALE) framework are used to solve the flow problem, which can be written as

Navier-Stokes equation

(1) $$\rho_{f}\frac{\partial v}{\partial t}{|}_{x}-2\mu_{f}\nabla \times \nabla^{s}+\rho (v-v^{m}\times \nabla v)+\nabla p=\rho b^{f}\;\mathrm{in}\;\Omega^{f}\times \left(0,T\right),$$

and the equation of continuity for incompressible flow is given by

(2) $$\nabla \times \vec{v}=0,$$

where $\rho_{f}$ denotes fluid density, $\mu_{f}$ is the viscosity, *v* is the velocity and *p* is pressure. $v^{m}$ and $b^{f}$ represent the mesh velocity and body force vector, respectively. The fluid domain is indicated as $\Omega^{f}$, and the time interval is denoted between (0,T). The time derivative of ALE mesh is represented as $\frac{\partial_{v}}{\partial_{t}}{|}_{x}$. The symmetric tensor $\nabla^{s}{v=(\nabla v+\left(\nabla v\right)}^{T}/2$ is called rate-of-deformation tensor.

For the present problem, an incompressible, viscous, constant-input mass flow rate of the fluid is applied between 4 to 12 μL/min in the inlet of the microfluidic valve, and the behavior of the structural membrane is analyzed. Water is considered as the working fluid, as shown in Table 1. Using the valve dimensions mentioned in Table 1 and the boundary conditions of the microfluidic device, the Reynolds number is calculated as

(3) $$Re=\frac{\rho (vD_{h})}{\mu }\gg 2300.$$

Thus, Reynolds number of the fluid flow inside a microfluidic valve is predicted before the analysis, and the flow is considered to be laminar throughout the analysis.

#### 3.1.2. Solid Formulation

The conservation law of linear momentum for a solid continuum is given with respect to spatial coordinate *x* as

(4) $$\frac{\partial^{2}u}{\partial t^{2}}=\nabla \left(FS^{s}\right)+\rho_{s}b^{s}\;\mathrm{in}\;\Omega^{s}\times \left(0,T\right),$$

where $\rho_{s}$, *u*, $b^{s}$ and $\Omega^{s}$ are the solid density, displacement field, body force vector, and solid domain, respectively. F=I+∇u is the deformation gradient tensor.

#### 3.1.3. FSI Interface Conditions

The interface conditions between solid and fluid are

(5) $$\frac{\partial u^{s}}{\partial t}=v^{f}\;\mathrm{on}\;\delta \Omega^{fs}\times \left(0,T\right),$$

(6) $$\sigma^{s}.n+\sigma^{f}.n=0\;\mathrm{on}\;\delta \Omega^{fs}\times \left(0,T\right),$$

where *n* and $\delta \Omega^{fs}$ are the outer normal at the solid and the FSI interface, respectively.

#### 3.1.4. Moving Mesh

In the ALE approach to the fluid-structure interaction (FSI) problems, the pseudo-medium’s boundary conditions are the solid deformation in the FSI interface. The governing equation of the displacement of the fluid mesh nodes is written as

(7) $$\nabla .\sigma^{p-m}=0\;\mathrm{in}\;\Omega^{f}\times \left(0,T\right),$$

where $\sigma^{p-m}$ is the Cauchy stress tensor of the pseudo-medium [5]. For every boundary *i*, a Dirichlet boundary condition may be given:

(8) $${\left(u_{i}\right)}^{p-m}={\left(u_{i}\right)}^{b},$$

where $u_{b}$ is the boundary displacement vector.

### 3.2. FSI Coupling Method

**Figure 4:**
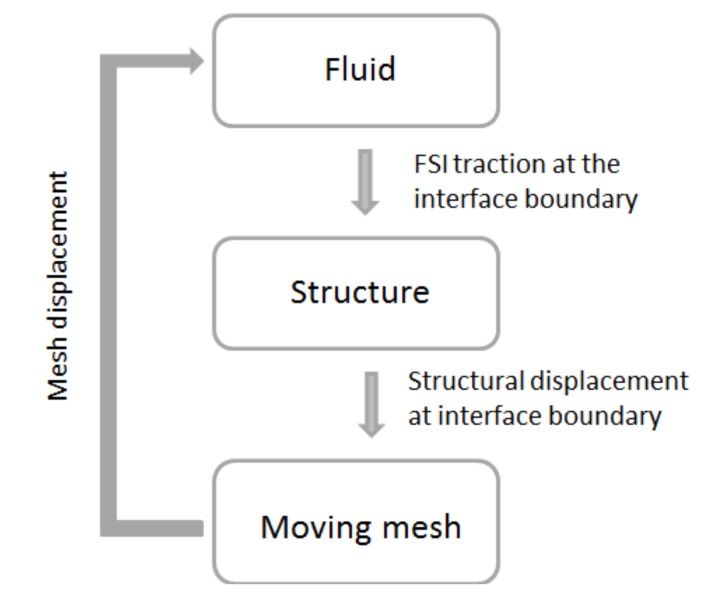
Schematic diagram of fluid-structure interaction.

FSI analysis of the two-way coupling method is illustrated in Figure 4. This coupling method is an iterative process: during each time step, both fields’ outcome data are transferred to another field, until convergence is achieved.

**Figure 5:**
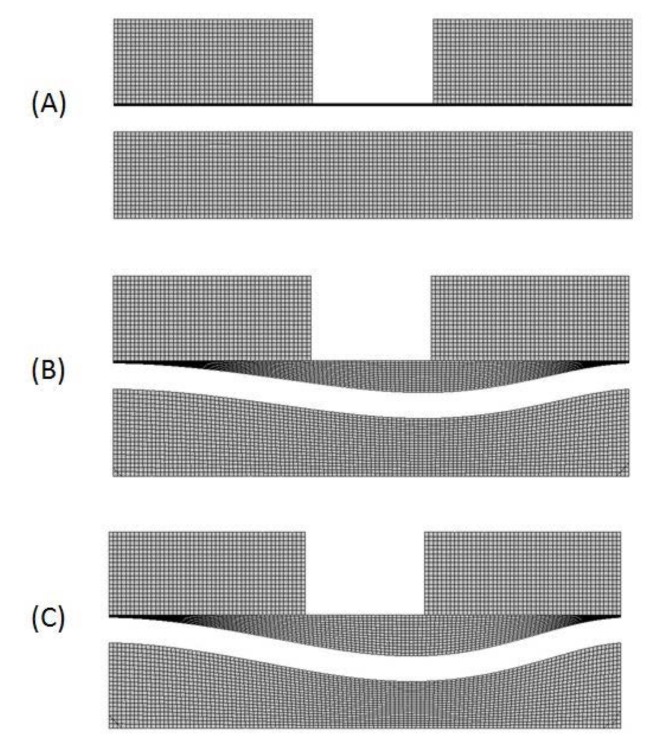
Initial and deformed stages of microvalve mesh in A-A’ cross-sectional plane: (**A**) Initial state of the microvalve. (**B**,**C**) Microvalve membrane deflected against pressure developed by fluid flow in the source region.

Initially, the fluid field is solved in ANSYS-CFX based on the given boundary conditions and are applied in the fluid domain. Later, the pressure from the fluid domain is transferred to the structural domain (ANSYS-Transient structure) through the coupling process. The transferred pressure is applied over the FSI interface surface in the structural model. Since external pressure is applied to the elastic structural membrane, it obtains a large deformation and changes its shape in the model. Furthermore, the deformed shape obtained from the solid domain sets the fluid mesh displacement equal to the solid domain deformation on the FSI interface (i.e., set $u^{mesh}=u^{solid}$). This step will update the Dirichlet boundary condition for the fluid solver. This process continues until it reaches convergence. The initial and deformed states of the microvalve model are shown in Figure 5.

In FSI analysis, converting from the open to closed state of the microvalve creates pressure instability and initiates the contact between the membrane surface and the wall. This contact happens mainly due to the low bending stiffness of the membrane. This type of contact analysis is not appropriate for a two-way FSI problem. This may be attributed to the instability and distortion in the mesh when membrane contacts with the wall during the two-way FSI analysis. Afrasiab et al. reported a similar problem related to this analysis [3,4]. Hence, in this study, the mass flow was fixed in the range of 4 μL/min to 12 μL/min to avoid membrane contact with the wall and the FSI analyses were performed.

The coupled fluid-structure model was solved by the commercial software ANSYS workbench. Finite volume in fluid and finite element in solids was solved in the ANSYS-CFX and ANSYS-Transient structure, respectively. Hexahedral elements were used for both domains and the full Newton numerical method is used to solve this problem.

## 4. Results and Discussion

### 4.1. Validation of FSI Results with Experimental Results

**Figure 6:**
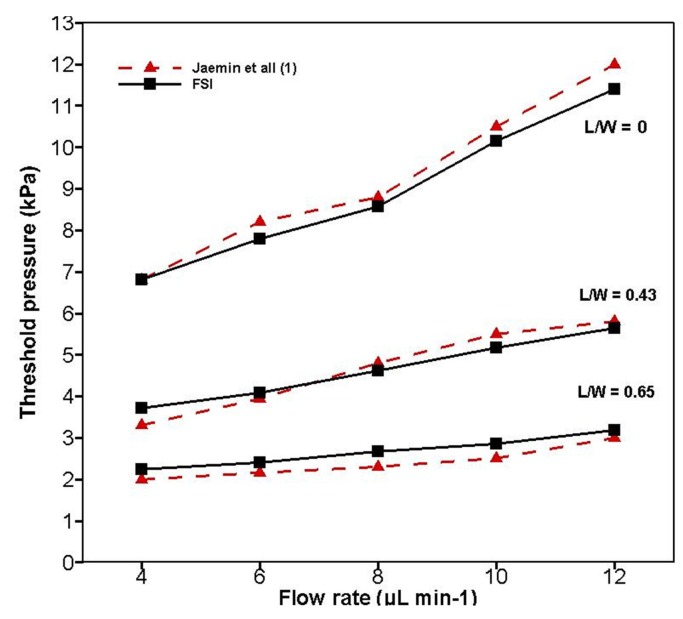
Comparison of experimental results with fluid-structure interaction (FSI) results for changes in opening threshold pressure against change in flow rate.

To achieve a low constant opening pressure to deflect the membrane and transform from the closed state to the open state, various L/W-ratio valve models were used in an FSI simulation. Furthermore, the FSI simulation results were compared and validated with Jaemin et al., as shown in Figure 6. In the FSI analysis, PDMS material was selected for the structural membrane, and water was selected as the fluid model. Their properties are listed in Table 2 and Table 3, respectively.

It was observed from Figure 6 that variations in the L/W ratio alter the threshold pressure opening and lead to a larger membrane deformation at the source region. Briefly, a higher L/W ratio increases the surface area of the membrane and volume at the source region, thereby leading to a rapid deformation of the membrane and an easy opening of the valve at minimum pressure. By contrast, with a lower L/W ratio, the volumes of the source and drain regions are equally separated, thus reducing the surface area in the source region of lower L/W ratio models.

### 4.2. Microvalve’s Membrane Surface Pressure for Various Geometries

**Figure 7:**
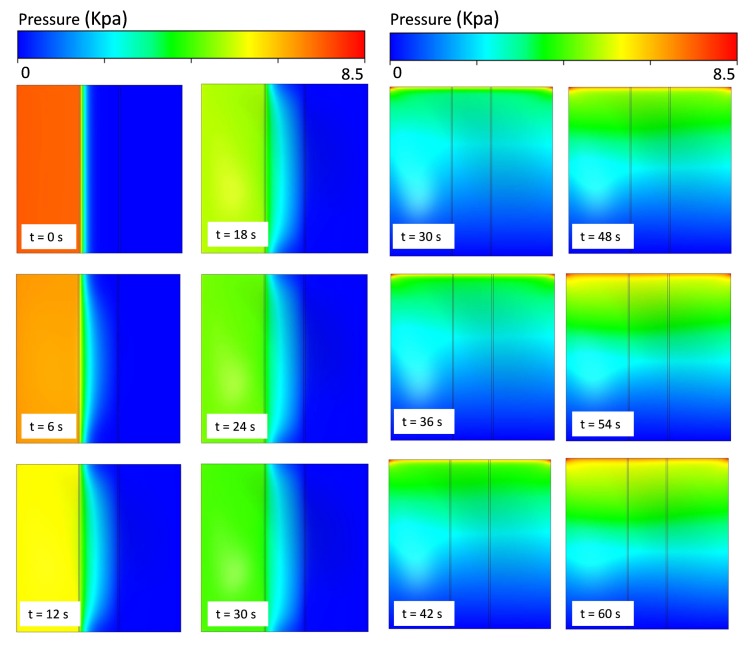
Pressure distribution for various time steps for L/W = 0 model in upper surfaces and lower surfaces of membranes in microvalves.

**Figure 8:**
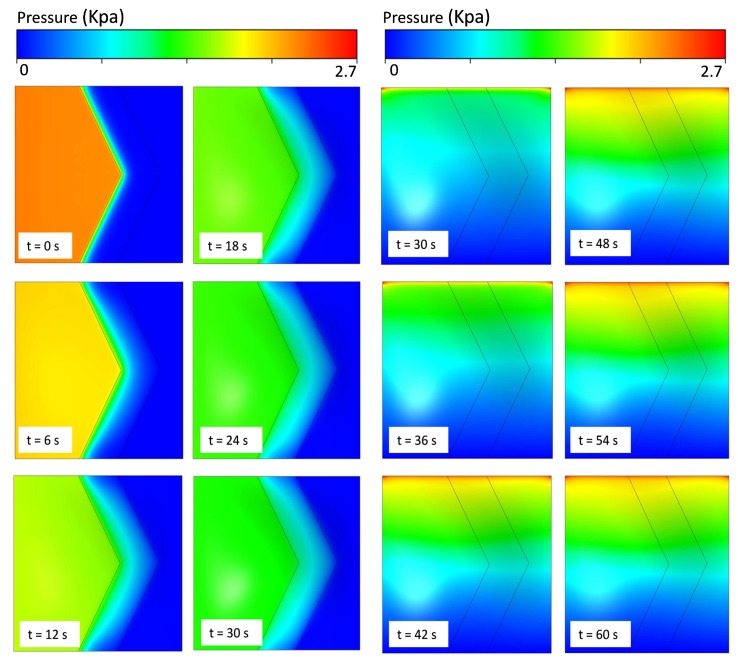
Pressure distribution for various time steps for L/W = 0.65 model in upper surfaces and lower surfaces of membrane in microvalves.

In order to evaluate the performance and behavior of the microvalve, different types of L/W-ratio models of microvalve geometry are used in an FSI analysis. In this FSI analysis, similar boundary conditions and an input flow rate of 8 μL/min are used in every L/W-ratio model to determine the pressure distribution of that model. The suitable results for L/W ratios of 0 and 0.65 are given below. In Figure 7 and Figure 8, the pressure distributions for the upper and lower surfaces of the membrane in 0-L/W-ratio and 0.65-L/W-ratio models are shown. As mentioned before, during the inlet conditions from 0–30 s, the fluid flows through the top region of the microvalve. From 30–60 s, the fluid flows in the bottom region of the microvalve. Figure 7 and Figure 8 show the pressure distribution from 0–30 s (upper surface of the membrane) and from 30–60 s (lower surface of the membrane).

As shown in Figure 7 and Figure 8, for both L/W-ratio models, during the initial time, the microvalve exhibits pressure development in the source region that is high. As time moves on, the pressure over the membrane surface becomes constant because at that time the membrane exhibits deflection, and a constant fluid flow is achieved. If constant pressure is reached over the surface of the membrane, then the membrane deflection also becomes stable. However, to achieve a quick deflection in the structural membrane, changes in the L/W ratio are used. Therefore, this area distribution leads to deformations in the membrane more rapidly for models with higher L/W ratios than those with lower L/W ratios. Thus, higher-L/W-ratio models require low pressure to deflect the membrane.

### 4.3. Velocity Contour Fields and Streamlines for Various L/W Ratio Models

**Figure 9:**
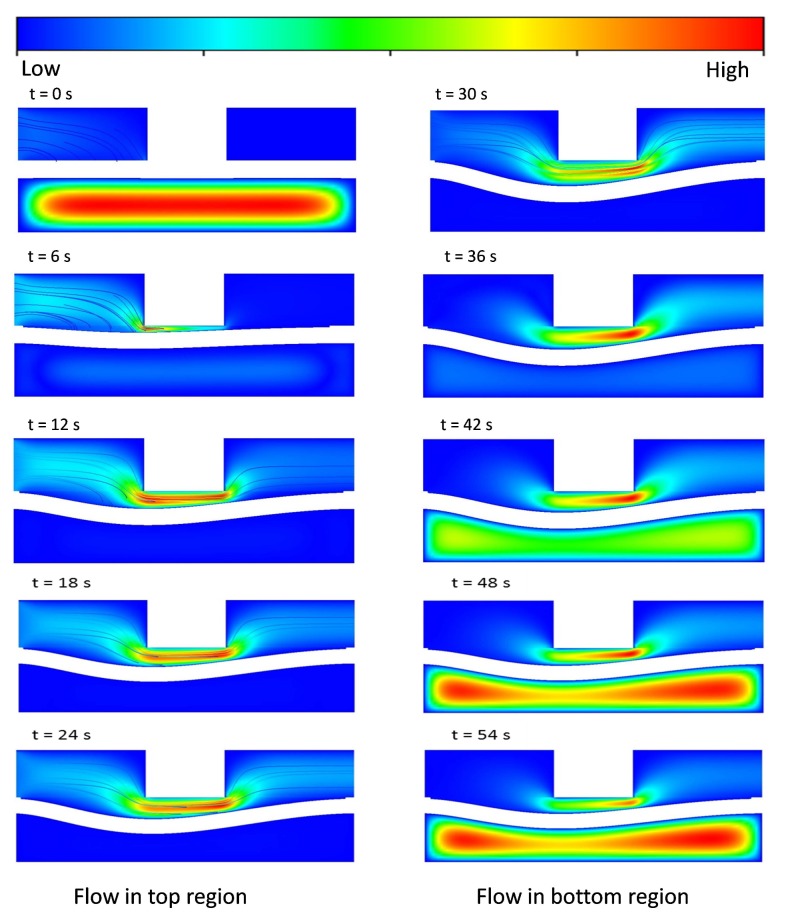
Velocity contours’ fields and streamlines for various time steps for L/W = 0 model in the A-A’ cross-sectional plane.

**Figure 10:**
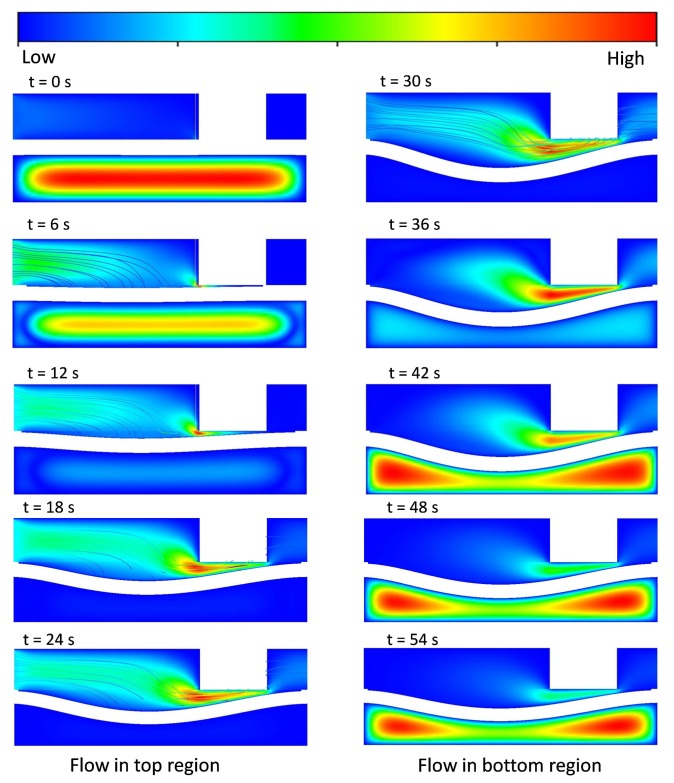
Velocity contours’ fields and streamlines for various time steps for L/W = 0.65 model in the A-A’ cross-sectional plane.

To investigate the behavior of the fluid flow inside the microvalve and the amount of structural membrane deflection, various L/W ratio models were used to perform an FSI analysis. In this FSI analysis, similar boundary conditions and an input flow rate of 8 μL/min were used in both 0-L/W-ratio and 0.65-L/W-ratio models to determine the velocity contour fields and streamlines, and the amount of membrane deformation of these models. The results for both cases are represented in the A-A’ cross-sectional plane and are depicted in Figure 9 and Figure 10. According to the changes in volume and surface area of the membrane in the source and drain for various L/W-ratio models, the flow and behavior of the structural membrane also varies, as shown in Figure 9 and Figure 10.

The results are shown for one complete cycle of 0–60 s. We can clearly visualize from 0–30 s the flow from the inlet in the top region of the microvalve. The structural membrane continues deflecting until we reach 30 s of fluid flow. This process is the open state of the membrane. After that, the top-region flow is stopped, and flow starts from 30 s at the bottom region. Because of the lower pressure in the structural membrane, it starts to deflect upwards. At a certain point in time, the deflected membrane contacts with the wall. This process is the closed state of the microvalve. A comparison of these two L/W-ratio models clearly shows that an increase in the L/W ratio changes the flow behavior of the fluid flow. Similarly, an increase in pressure in the source region and a large deformation of the membrane are also achieved.

### 4.4. Structural Membrane Deflection against Fluid Flow

**Figure 11:**
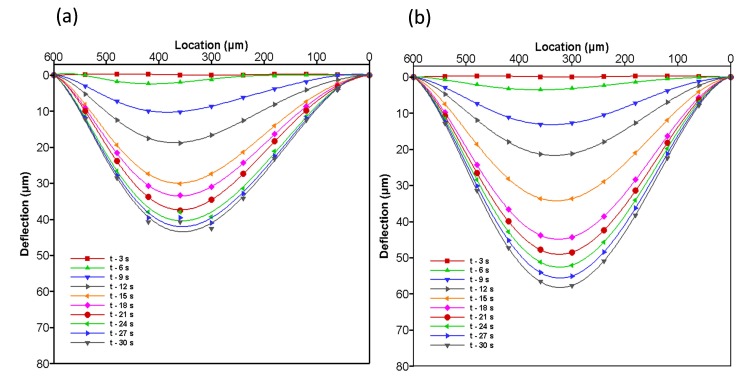
Deformation of membrane in the A-A’ cross-sectional plane is shown in various time steps at an input flow rate of 8 μL/min are used in both L/W-ratio models (**a**) L/W = 0 and (**b**) L/W = 0.65 model.

To study the behavior of the structural membrane of the microvalve, two L/W-ratio models are compared. These are shown in Figure 11. Similar boundary conditions and an input flow rate of 8 μL/min are used in both L/W-ratio models to determine the effect of the deflection of the membrane against the fluid flow. The behaviors of membrane deflection for both L/W-ratio models are shown in the A-A’ cross-sectional plane of the structural membrane in Figure 11.

As mentioned in previous sections, membrane contact with the wall leads to instability and distortion in mesh of FSI . Therefore, these analyses are performed without the contact problem. In Figure 11, we can easily visualize the behavior of the membrane in the A-A’ cross-sectional location of the structural membrane for both L/W-ratio models at various time steps between 0–30 s.

The graph indicates that initially the membrane begins to deform in a step-by-step manner. After a certain time, the deformation becomes constant because the fluid flow becomes constant (constantly flows from the source to the drain region). In lower-L/W-ratio models, the volume of the source region is limited. Hence, the model cannot produce higher pressure and creates a large deflection in the membrane. Even when compared with that at final deformation, the membrane deflection magnitude is lower in lower-L/W-ratio models than in higher-L/W-ratio models. The initial and total deformation of the membrane increase in higher-L/W-ratio models. These results indicate that increasing the L/W ratio of the model increases the initial and total deformation of the membrane.

### 4.5. Effects of Change in Material Properties of the Structure

**Figure 12:**
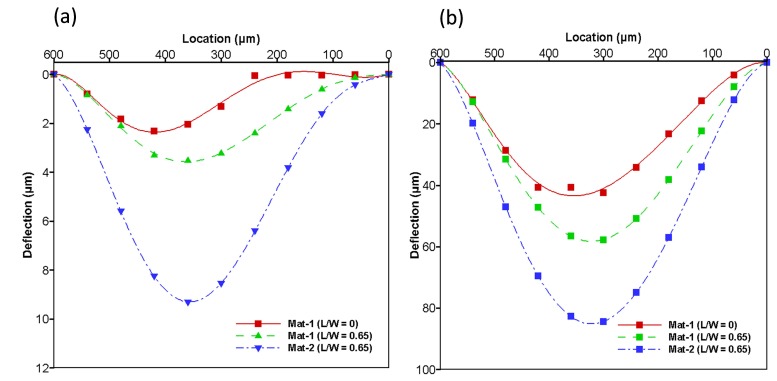
Deformation of membrane in the A-A’ cross-sectional plane is shown in various material properties at an input flow rate of 8 μL/min are used in both L/W-ratio models (**a**) L/W = 0 and (**b**) L/W = 0.65 model.

PDMS is one of the most-used materials in nano-electromechanical systems (NEMS) and micro-electomechanical systems (MEMS) because of its elastic properties. Varying the amount of crosslinker in a PDMS network changes the stiffness of the material [20]. In order to study this parameter and its effect on the structural membrane, we change the material properties. The results are shown in Figure 12. In this figure, the initial and final deformation at 6 and 30 s are shown in Figure 12a,b, respectively. The results clearly indicate that changes in the material properties of the structural membrane at L/W = 0.65 cause an increase in the amount of deformation in both the initial and final stages of the structural membrane. This result suggests that changes in material properties impact the structural deformation and behavior against fluid flow.

### 4.6. Effects of Change in Inlet Height

**Figure 13:**
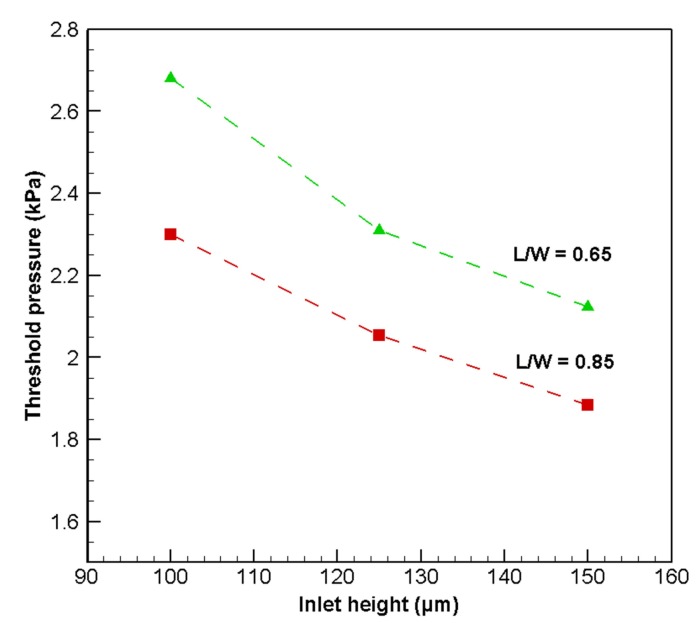
Opening threshold pressure of microvalve for various inlet region heights for L/W = 0.65 and 0.85 models.

In order to check the parameter of the change in inlet height, three different inlet region heights are selected and analyzed. The results are shown in Figure 13. Throughout the analysis, the outlet region height is fixed at 100 μm. The figure clearly shows that a greater inlet region height reduces the pressure required to open the membrane.

## 5. Conclusions

The complex fluid-structure interaction between deformable microvalve structural membrane behavior against the fluid flow inside the valves was studied using an FSI model. Using the model, it was observed that an increase in L/W ratio increases the volume in the source region and decreases the volume in the drain region of the microvalve. In addition, an increase in L/W ratio separates the membrane surface area within those regions. Interestingly, the separation completely alters the nature of fluid flow and the behavior of membrane deflection inside the microvalve.

Compared with lower-L/W-ratio models, higher-L/W-ratio models exhibit better performance in terms of membrane deflection and low opening threshold pressure. A higher-L/W-ratio model favors a higher volume in the source region, which enables more rapid structural membrane deflection than the lower L/W ratio models. L/W = 0.65 or higher ratio models can deflect the membrane in lower pressure regime favoring the fluid flow than other lower ratio models.

Finally, using an FSI simulation, selected parameters such as the material properties of the structural membrane and the inlet region height were analyzed for better rapid deflection and low opening threshold pressure. This creates a new step in understanding better fluid flow inside a microvalve. 

## Figures and Tables

**Table 1:** Dimensions of geometry.

Design Parameters	Dimensions (μm)
Microvalve width	600
Microvalve length	600
Microvalve top layer	100
Microvalve bottom layer	100
Microvalve seat width	140
Microvalve thickness	30

**Table 2:** Physical properties of solid.

Materials	Density (kg/m${}^{3}$)	Elastic Modulus (MPa)	Poisson’s Ratio
Material-1 (Polydimethylsiloxane (PDMS))	960	3.5	0.49
Material-2 (Polydimethylsiloxane (PDMS))	960	2	0.49

**Table 3:** Physical properties of fluid.

Materials	Density (kg/m${}^{3}$)	Viscosity (kg/m·s)
Water	1000	0.001

## References

[1] Whitesides G.M. (2006). The origins and future of microfluidics. Nature.

[2] Chakraborty D., Prakash J.R., Friend J., Yeo L. (2012). Fluid-structure interaction in deformable microchannels. Phys. Fluids.

[3] Afrashib H., Movahhedy M.R., Assempour A. (2011). Finite element and analytical fluid-structure interaction analysis of the pneumatically actuated diaphragm microvalues. Acta Mech..

[4] Yang F., Kao I. (2000). Analysis of fluid flow and deflection for pressure-balanced MEMS diaphragm valves. Sens. Actuators.

[5] Afrasiab H., Movahhedy M.R., Assempour A. (2011). Fluid-structure interaction analysis in microfluidic devices: A dimensionless finite element approach. Int. J. Numer. Methods Fluids.

[6] Mitchell M.C., Spikmans V., Manz A., de Mello A.J. (2001). Microchip-based synthesis and total analysis systems (*μ*SYNTAS): Chemical microprocessing for generation and analysis of compound libraries. J. Chem. Soc. Perkin Trans..

[7] Powers M.J., Domansky K., Kaazempur-Mofrad M.R., Kalezi A., Capitano A., Upadhyaya A., Kurzawski P., Wack K.E., Stolz D.B., Kamm R. (2002). A microfabricated array bioreactor for perfused 3D liver culture. Biotechnol. Bioeng..

[8] Cho S.W., Kang D.K., Choo J.B., Demllo A.J., Chang S.I. (2011). Recent advances in microfluidic technologies for biochemistry and molecular biology. BMB Rep..

[9] Thorsen T., Maerkl S.J., Quake S.R. (2002). Microfluidic large-scale integration. Science.

[10] Lee C.-C., Sui G., Elizarov A., Shu C.J., Shin Y.S., Dooley A.N., Huang J., Daridon A., Wyatt P., Stout D. (2005). Multistep synthesis of a radiolabeled imaging probe using integrated microfluidics. Science.

[11] Shin J., Park H., Dang V.B., Kim C.W., Kim S.J. (2015). Elastometric microfluidic valve with low, constant opening threshold pressure. RSC Adv..

[12] Grover W.H., Skelley A.M., Liu C.N., Lagally E.T., Mathies R.A. (2003). Monolithic memberane valves and diaphragm pumps for practical large scale integration into glass microfluidic devices. Sens. Actuvators B.

[13] Kartalov E.P., Scherer A., Quake S.R., Taylor C.R., Anderson W.F. (2007). Experimentally validated quantitative linear model for the device physics of elastometric microfluidic valves. J. Appl. Phys..

[14] Mohan R., Schudel B.R., Desai A.V., Yearsley J.D., Apblett C.A., Kenis P.J. (2011). Design considerations for elastometric normally closed microfluidic valves. Sens. Actuvators B.

[15] Sun H., Li Z., Tao J. 3D simulation of a microchip using Finite element method. Proceedings of the 14 IFToMM World Congress.

[16] (2015). The ANSYS User Guide.

[17] Johnson A.A., Tezduyar T.E. (1999). Advanced mesh generation and update method for 3D flow simulations. Comput. Mech..

[18] Ryzhakov P.B., Oñate E. (2017). A finite element model for fluid-structure interaction problems involving closed membranes, internal and external fluids. Comput. Methods Appl. Mech. Eng..

[19] Slone A.K., Pericleous K., Bailey C., Cross M., Bennett C. (2004). A finite volume unstructured mesh approach to dynamic fluid-structure interaction: An assessment of the challenge of predicting the onset of flutter. Appl. Math. Model..

[20] Wang Z., Volinsky A.A., Gallant N.D. (2014). Crosslinking effect on polydimethylsioxane elastic modulus measured by custom-built compression instrument. J. Appl. Polym. Sci..

